# The effects of remimazolam combined with sufentanil on respiration, circulation and sedation level in patients undergoing colonoscopy

**DOI:** 10.1186/s12871-024-02644-0

**Published:** 2024-07-25

**Authors:** Qirui Sun, Jiating Cheng, Weiping Lei, Xinlei Lu, Yaqin Huang, Jianliang Sun

**Affiliations:** 1 Department of Anesthesiology, Chengbei Branch of Hangzhou First People’s Hospital (Hangzhou Geriatric Hospital), Hangzhou, 310000 China; 2 Department of Anesthesiology, Affiliated Hangzhou First People’s Hospital, School of Medicine, Westlake University, Hangzhou, 310000 China; 3 School of Second Clinical Medical College, Wenzhou Medical University, Wenzhou, 310053 Zhejiang China; 4 Department of Anesthesiology, Sir Run Run Shaw Hospital, School of Medicine, Zhejiang University, Hangzhou, 310016 China

**Keywords:** Remimazolam, Sufentanil, Propofol, Colonoscopy, Sedation

## Abstract

**Background:**

The main sedative which is propofol in painless gastroenteroscopy, has a high risk of reducing blood pressure and respiratory depression. Remimazolam (a short-acting benzodiazepine) is expected to be widely used in painless gastroenteroscopy due to its rapid onset, rapid metabolism and light respiratory and circulation inhibition.

**Methods:**

A randomized, single-blind, parallel, controlled study, 123 outpatients who were undergoing painless colonoscopy and ramdomly divided into group A, B and C, in Hangzhou First People’s Hospital, July-December 2021. All patients were intravenously injected with 5 µg sufentanil for analgesic preconditioning. The group A was induced by 0.2 mg/kg remimazolam besylate. The group B was induced by 0.25 mg/kg remimazolam besylate. And the group C was inducted by 2.0 mg /kg propofol. If the patients had limb movement or MOAA/S score > 3 and so on, remimazolam besylate was added at 2.5 mg/ time in group A and B, and propofol emulsion injection was added at 0.5 mg/kg/ time in group C. During the operation, according to the actual situation, remimazolam was per added 2.5 mg in the experimental group, and propofol was 0.5 mg/kg in the control group. Heart rate (HR), non-invasive blood pressure (BP), respiratory rate (RR), pulse oxygen saturation (SpO_2_), and improved vigilance/sedation score (MOAA/S) of patients was recorded from entering endoscopy room to get out of the anesthesia recovery room, also including perioperative adverse events, other medications or treatments, the time of patients waking up and leaving the hospital.

**Results:**

The successful rate of induction in three groups was 100%. There was no significant difference in the sedation completion rate among the three groups (Group A:90.2%, Group B: 92.7%, Group C: 92.7%, *P* = 1.000). The rate of adverse events after administration: group A(27.0%) and B(36.8%) both lower than group C(71.0%),*P* < 0.001;There was no significant difference between group A and group B, *P* > 0.744;The average time from the last drug administration to meet the discharge criteria of the subjects in three groups was as follows: The average time of group A(16.2 min) and Group B(16.5 min) both shorter than group C(19.6 min), *P* = 0.001; There was no significant difference between group A and group B, *P* = 0.742. Conclusions: This study revealed that remimazolam is a safe and effective medication for colonoscopy sedation, the security of remimazolam is better than propofol, and the sedative effect with the initial dose of 0.25 mg/kg of remimazolam is optimal.

**Trial registration:**

China Clinical Trial Center with registration number: 2100052615,02/11/2021.

## Background

Endoscopy is a well-accepted and widely used method in the diagnosis and treatment of gastrointestinal diseases [1, 2]. Sedation and anesthesia in endoscopic procedures has the benefit of eliminating anxiety and discomfort in patients and improving patient acceptance of and satisfaction with endoscopic procedures, it’s also useful for improving the completion rate, quality of endoscopic examination and treatment outcomes of therapeutic endoscopy from the viewpoint of endoscopists [3].

In China, the total intravenous anesthesia(TIVA) commonly used for sedating patients during a procedure involves the use of two major hypnotics, midazolam, and propofol, often in combination with an opioid analgesic, typically fentanyl, or sufentanil [4, 5]. Midazolam has potent anxiolytic effect with amnesia, sedation, skeletal muscle relaxant activity, and good hemodynamic stability with lack of significant side effects in doses < 0.5 mg/kg and the onset time is about 3–5 min. A single dose of midazolam with an elimination half-life of 20–80 min, while multiple doses produced greater accumulative effects of its long-acting metabolite that causes a slower recovery of neuropsychiatric function [6, 7]. Propofol is a commonly used agent in total intravenous anesthesia. Propofol has high lipophilicity and can quickly cross the blood-brain barrier to achieve a deep sedative effect in a short period of time. However, propofol has a number of known limitations, such as high incidence of hypotension, respiratory depression, pain on injection and a lack of availability of antagonists [4, 8].

Remimazolam (CNS7056) is a new ultrashort-acting benzodiazepine developed for use in sedation and anesthesia that acts on the central GABA_a_ receptor, opening the channel and increasing the inward flow of chloride ions, causing hyperpolarization of the nerve cell membrane and thus inhibiting neuronal activity, producing sedation and amnesia etc [9, 10]. Phase I pharmacokinetic trials demonstrated that remimazolam had an onset time of 1–3 min and a steady-state half-life of 7–8 min after a 2-h simulated infusion similar to propofol, and there is no active metabolite and almost no accumulation [11–13]. When compared with propofol in both sedation and general anesthesia, remimazolam exhibited better safety profile, including a lower incidence of hypotension, less bradycardia treatment requirement, and no pain on injection [14, 15], and flumazenil can antagonize the effects of remimazolam there is almost no rebound phenomenon [12, 16].

The study used a randomized, single blind, controlled method to evaluate the safety and effectiveness of remimazolam in colonoscopy diagnosis and treatment, and remimazolam is divided into two dose groups for administration to find a suitable administration scheme.

## Methods

### General information

This study was approved by the Ethics Committee of Hangzhou First People’s Hospital affiliated to Zhejiang University school of medicine (Approval number: 2020 YLS No. (041) – 01,24/12/2020). All subjects were fully informed about the experimental protocol and voluntarily signed an informed consent form before the start of the study. The inclusion, exclusion and abscission criteria are shown in Table 1.

**Table 1:** Study inclusion and exclusion criteria

**Inclusion criteria**
The American Anesthesiologists Association (ASA) was Grade I or Grade II;
Body mass index (BMI) 18∼28 kg /m^2^;
Age: 18–65;
Patients undergoing routine colonoscopy.
**Exclusion criteria**
Pregnant or lactating women and patients (including men) with birth plans within 3 months;
Patients with obvious respiratory and circulatory dysfunction, abnormal blood routine and blood biochemical indexes before operation;
People suffering from serious neuropsychiatric diseases;
Take benzodiazepines or opioids intermittently in the last three months or every day within one month;
Allergies or contraindications to benzodiazepines, opioids or drugs used in this study and their drug components;
Patients who were judged to have difficulty in respiratory tract management: improved Markov classification III and above;
Before anesthesia induction, BIS<90.
**Abscission criteria**
Regardless of the time period and reason, as long as the observation cycle involved in the research scheme is not completed, it is considered as an abscission case;
In the process of sedation, remimazolam was changed to propofol to complete the operation.

### Scoring standard

Sedation level was assessed using a 6-point sedation scale, which was modified from the observer assessment of alertness and sedation scale (MOAA/S) (Table 2). The incidence and severity of injection pain were assessed using a four point scale 0 = no pain; 1 = mild pain; 2 = moderate pain and 3 = severe pain [17]. The modified Aldrete score (Table 3) was used to evaluate whether the patient could leave the Post -Anesthesia Care Unit (PACU). Recovery from sedation was assessed by using a modified Aldrete scoring system, which was evaluated every 3 min after the removal of the endoscope [18, 19].

**Table 2:** Description of modified observer’s assessment of alertness/ sedation scores

Score	Description
5	Responds readily to name spoken in normal tone
4	Lethargic response to name spoken in normal tone
3	Responds only after name is called loudly and/or repeatedly
2	Responds only after mild prodding or shaking
1	Responds only after painful trapezius squeeze
0	No response after painful trapezius squeeze

**Table 3:** Modified Aldrete score

Parameters	Description of the patient	Score
Activity level	Moves all extremities voluntarily/on command	2
	Moves 2 extremities	1
	Cannot move extremities	0
Respiration	Breathes deeply and coughs freely	2
	Is dyspneic, with shallow, limited breathing	1
	Is apneic	0
Circulation	Is 20 mmHg > preanesthetic level	2
	Is 20 to 50 mmHg > preanesthetic level	1
	Is 50 mmHg > preanesthetic level	0
Consciousness	Is fully awake	2
	Is arousable on calling	1
	Is not responding	0
Oxygen saturation as determined by pulse oximetry	Has level > 90% when breathing room air	2
	Requires supplemental oxygen to maintain level > 90%	1
	Has level < 90% with oxygen supplementation	0

### Procedures (The trial procedure is shown in Fig. 1)

Subjects who met the inclusion criteria were randomly assigned to three groups in a ratio of 1:1:1, using the random number table generated by SPSS 26.0 (SPSS Inc. Chicago, IL, United States). Patients were asked to fast 4–6 h before the examination, and intestinal preparation (lactulose and magnesium sulfate) was used to reduce bowel movements [20]. After entering the Operation room, a “Venturi” mask with an oxygen flow of 2–4 L/min was used to inhale oxygen, open the venous access, connect the monitor, and continuously monitor the electrocardiogram (ECG), invasive blood pressure (INBP), respiratory rate (RR), pulse oxygen saturation (SpO_2_) and heart rate (HR). The MOAA /S score assessment and bispectral index (BIS) to evaluate the depth of sedation, and the MOAA /S score was selected as the primary outcome measure. One hundred and twenty-three patients were divided into three groups: remimazolam 0.2 mg/kg (Group A), 0.25 mg /kg (Group B), and propofol 2 mg/kg (Group C).

Patients received a single dose of sufentanil 5.0 µg for analgesia. According to grouping, patients were received remimazolam or propofol intravenously within 1 min after sufentanil administration for anesthesia. Evaluation was performed every 30 s after administering sedatives, and the evaluation interval was shortened to 5 s at the beginning of study drug administration until the MOAA/S score was ≤ 3, and the longest evaluation time was no more than 3 min after the beginning of study drug administration, otherwise, supplemental remimazolam2.5 mg or propofol 30 mg were administered, and failure was recorded if two additional attempts could not make MOAA/S score was ≤ 3. During the procedure, once the patients’ eyelash reflexes (detected 2-min interval)recovered, frown, limb movement, moaning or MOAA /S score >3, supplemental remimazolam 2.5 mg or propofol 30–50 mg were administered to maintain sedation. If the patient’s body movement still significantly affects the operation after two doses were administered consecutively (the interval >2 min), it is considered sedation failure, and other sedatives were used as the rescue drug to finish the operation.

In case of hypotension, defined as systolic blood pressure less than 80 mmHg or a fall in systolic or diastolic BP of 30% or more below baseline (The setting standard for hypotension was a systolic blood pressure of less than 90mmHg, and the standard for hypotension was adjusted to a systolic blood pressure of less than 80mmHg according to the patient’s body position, measurement site and clinical observation), a bolus of norepinephrine (10ug IV) or ephedrine (6 mg IV) was administered; in case of bradycardia, defined as HR < 50, a bolus of atropine was administered (0.5 mg). In case of SpO_2_ drops below 92%, chin lift and/or manual or mechanical ventilation. If the MOAA/S score < 5 when completion of colonoscopy 30 min, administrate flumazenil which is the antagonist benzodiazepines [16, 18, 19] .The main observation index and secondary observation index are listed in Table 4.

**Table 4:** Primary and secondary outcome variables

**Primary outcome measure**	
	The incidence rate of hypotension: the percentage of patients in each group with hypotension during anesthetic maintenance using target drugs (Remimazolam or propofol during clinical observation period).
**Secondary objectives**	
	Sedation completion rate: the percentage of people in each group who successfully induced anesthesia using target drugs (Remimazolam or propofol in this clinical observation) and did not use remedial drugs during anesthesia maintenance in the total number of people in each group.
	HR, NIBP, RR, SPO_2_, MOAA/S scores and BIS of T0 (at admission), T1 (1 min after administration), T2 (3 min after administration), T3 (5 min after administration), T4 (10 min after administration), T5 (when the patient wakes up);
	Adverse events during the sedation period, including hypertension, tachycardia, bradycardia, hypoxemia, dizziness, hiccup;
	The incidence of pain at the injection site, obvious body movement and obvious intestinal peristalsis;
	The time from the last administration to the full recovery of the subject;
	The time from the last administration to the time when the subject’s modified Aldrete score ≥ 9;
	Other drug usage.

### Sample size and power

The sample size was estimated on PASS software version 16.0. Based on a previous study, the incidence of hypotension with remimazolam in painless gastrointestinal endoscopic was 13.04%, while the incidence with propofol was 42.86%^(15)^. For a 1-sided type I error rate of 0.05 and a target power of 80%, 37 patients were required for the treatment group. In this study, the data from the completed sedation patients were used for analysis and statistics, and considering the failure rate of about 10%, 123 cases were finally included.

### Statistical methods

SPSS26.0 software was used for data analysis. Measurement data were expressed as mean ± standard deviation (Mean ± SD), and counting data were expressed as Χ ^2^ Test, *P*<0.05 is considered statistically significant, and all tests are bilateral tests.

**Fig. 1:**
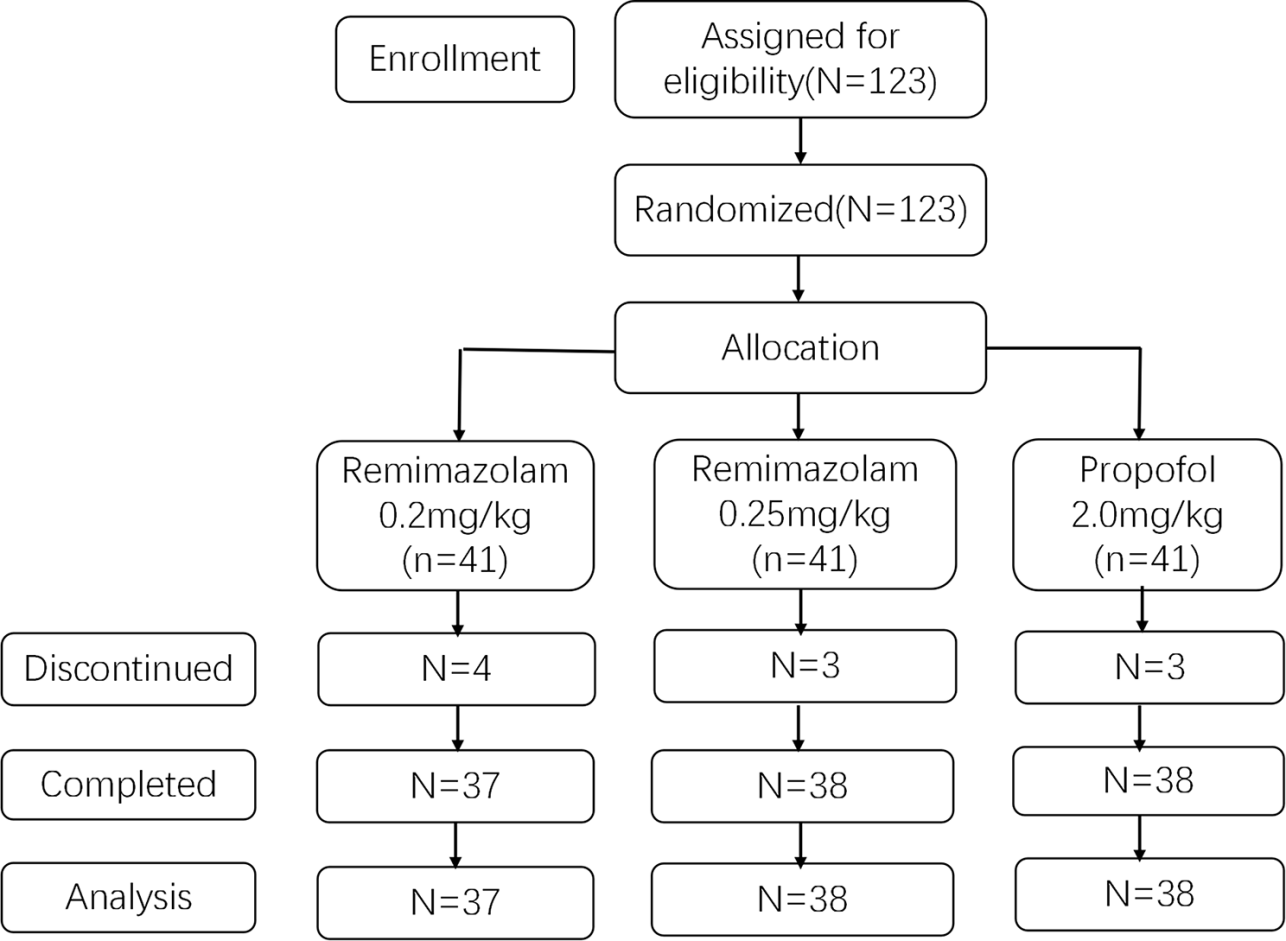
Flowchart of study patient enrollment

## Results

### General aspects and data

The subjects selected for the study were randomly divided into three groups, and each group consisted of 41 participants. The subjects were blind and did not know the trial grouping. In group A, 37 cases succeeded in sedation and 4 cases failed; In group B, 38 cases succeeded in sedation and 3 cases failed; In group C, sedation was successful in 38 cases and 3 cases failed. Finally, take 37 cases in group A, 38 cases in group B and 38 cases in group C to data analysis. There was no statistically significant difference in the baseline data of the subjects (Table 5). There was no significant difference in HR, BP, SpO_2_, RR, MOAA /S scores of the three groups before induction (Table 6).

**Table 5:** Demographics of the 3 study arms

		Group A *N* = 37, no. (%)	Group B *N* = 38, no. (%)	Group C *N* = 38, no. (%)	*P* value
Age, y	Mean	52.5	47.7	52.5	0.299
	SD	12.83	15.01	12.36	
Sex	Male	23(62.2%)	16(42.1%)	24(63.2%)	0.115
	Female	14(37.8%)	22(57.1%)	14(36.8%)	
Height, cm	Mean	166.0	163.4	165.5	0.227
	SD	6.50	6.29	8.20	
Weight, kg	Mean	63.3	60.7	62.4	0.579
	SD	9.72	8.87	13.22	
BMI, kg/m^2^	Mean	22.9	22.7	22.6	0.905
	SD	2.81	2.63	3.32	
ASA-PS score	I	29(78.4%)	31(81.6%)	29(76.3%)	0.852
	II	8(21.6%)	7(18.4%)	9(23.7%)	
Operation time	Mean	19.5	18.0	20.9	0.496
	SD	12.25	9.23	10.72	

**Table 6:** Baseline values of vital signs

		Group A(*N* = 37)	Group B(*N* = 38)	Group C(*N* = 38)	*P* value
Heart rate, (beats/min)	Mean	75.0	75.5	72.4	0.365
	SD	14.82	13.27	13.59	
SBP (mmHg)	Mean	130.5	128.0	127.0	0.733
	SD	21.81	19.20	19.21	
DBP (mmHg)	Mean	73.2	69.6	72.5	0.273
	SD	12.85	12.22	1063	
MAP (mmHg)	Mean	92.4	89.1	90.6	0.559
	SD	14.65	12.37	12.53	
SPO_2_(%)	Mean	99.5	99.3	99.4	0.250
	SD	0.80	0.94	0.79	
Respiratory rate (breaths/minute)	Mean	17.8	17.1	16.7	0.103
	SD	3.32	3.23	3.21	
MOAA/S score	Mean	5.0	5.0	5.0	1.000
	SD	0	0	0	
BIS	Mean	95.4	94.8	94.4	0.784
	SD	2.07	3.11	1.14	

### Vital signs during sedation

**Fig. 2:**
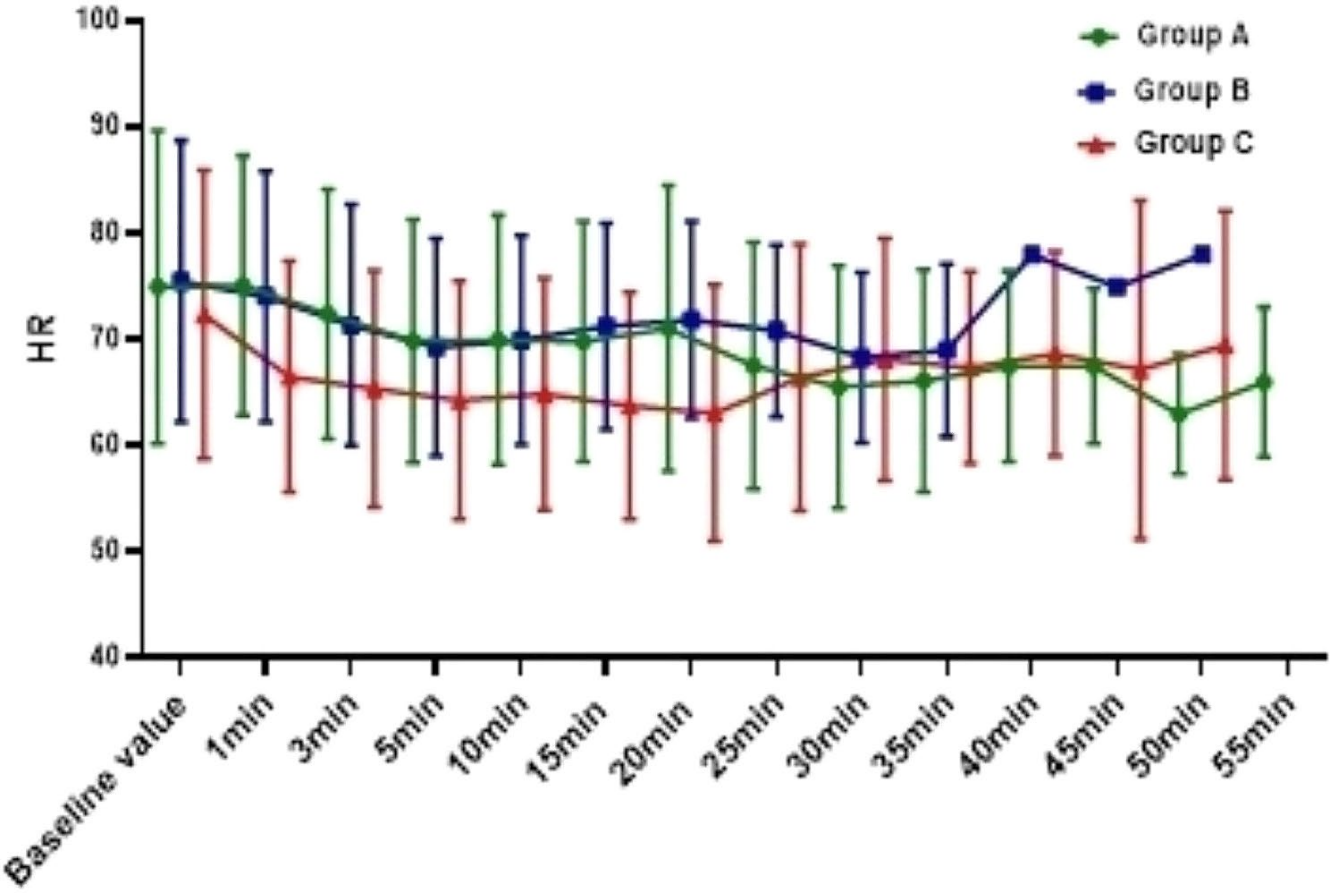
The change trend of HR at each time point during the perioperative period was compared among the three groups

**Fig. 3:**
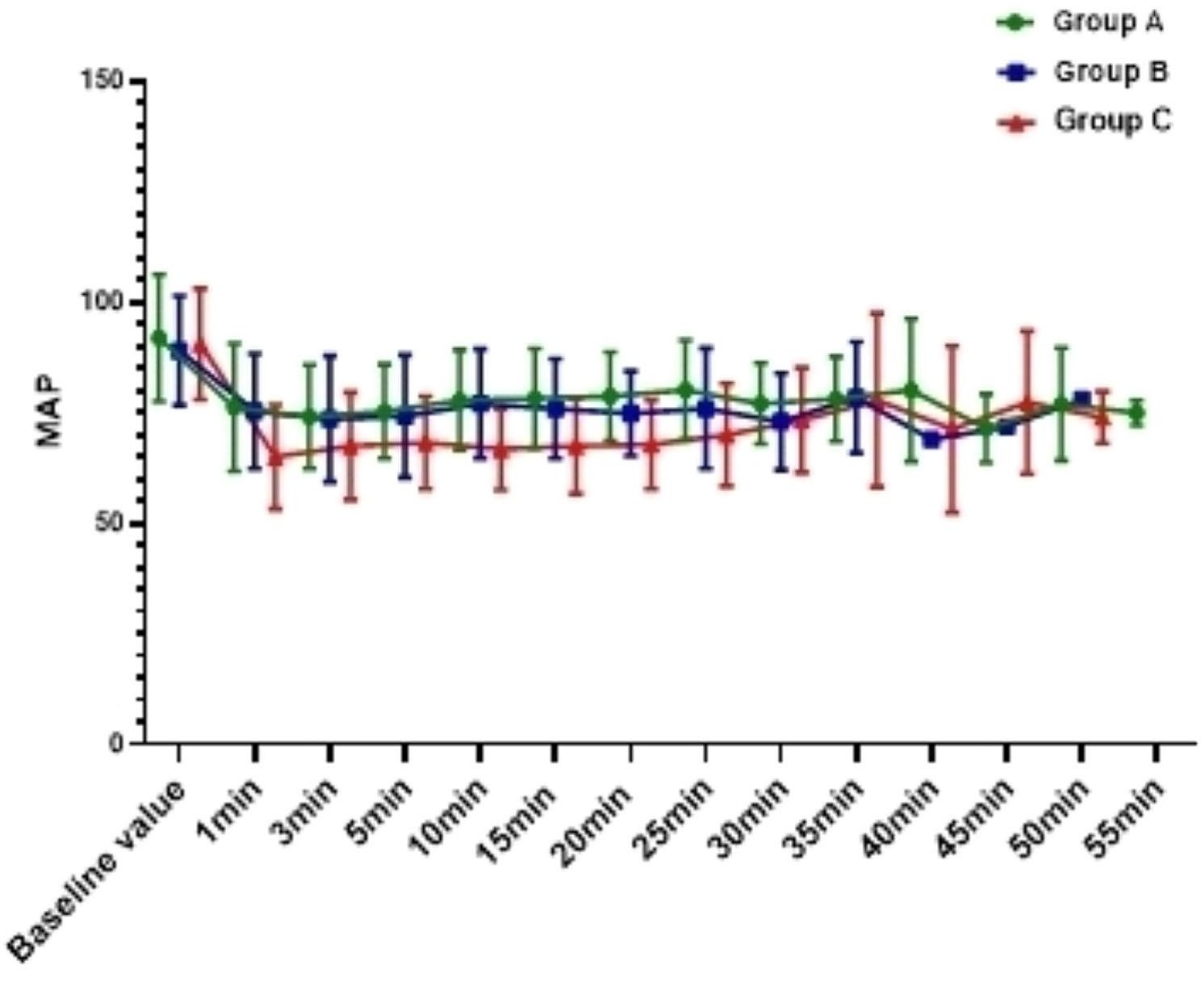
The change trend of MAP at each time point during the perioperative period was compared among the three groups

**Fig. 4:**
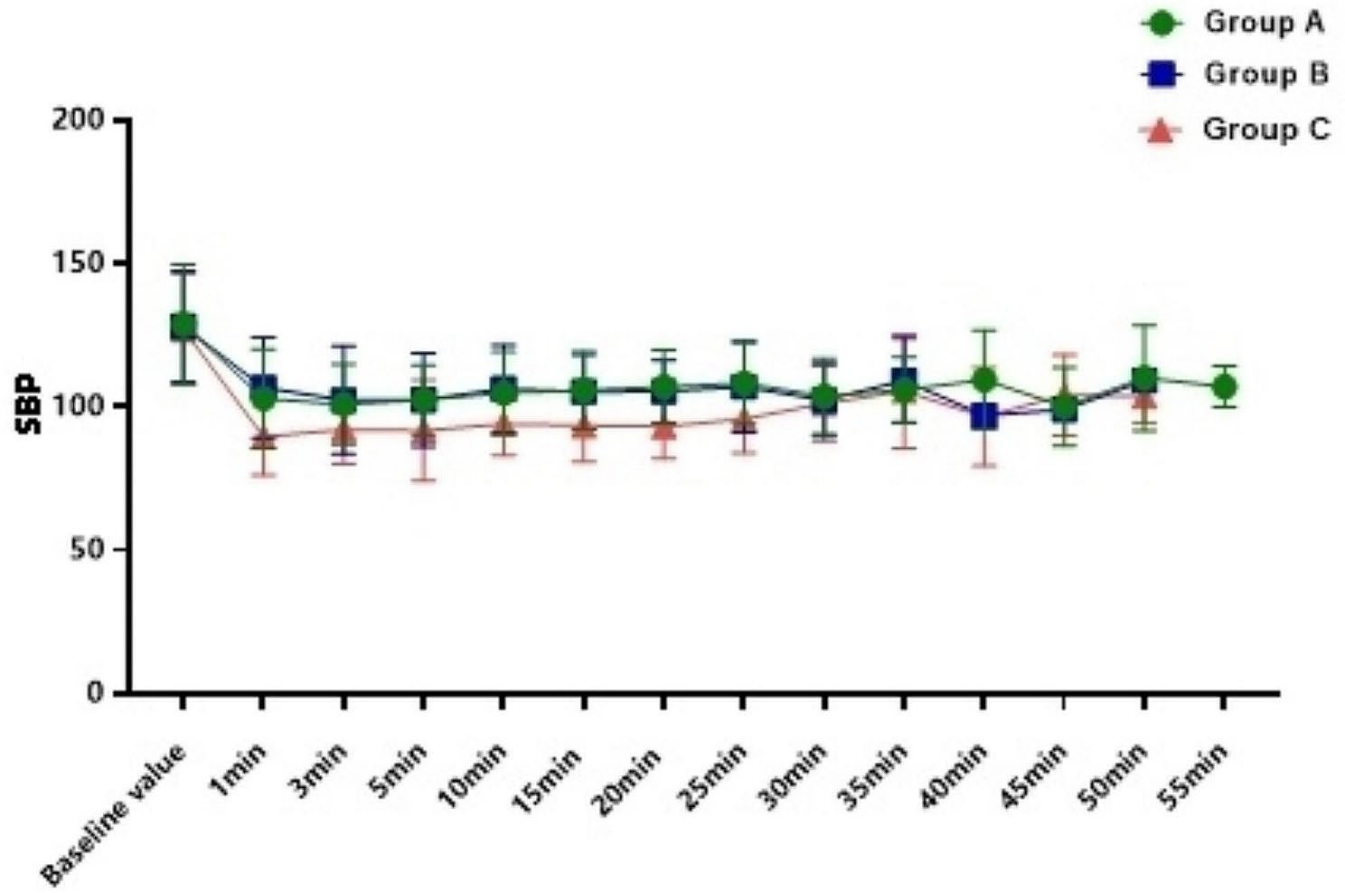
The change trend of SBP at each time point during the perioperative period was compared among the three groups

**Fig. 5:**
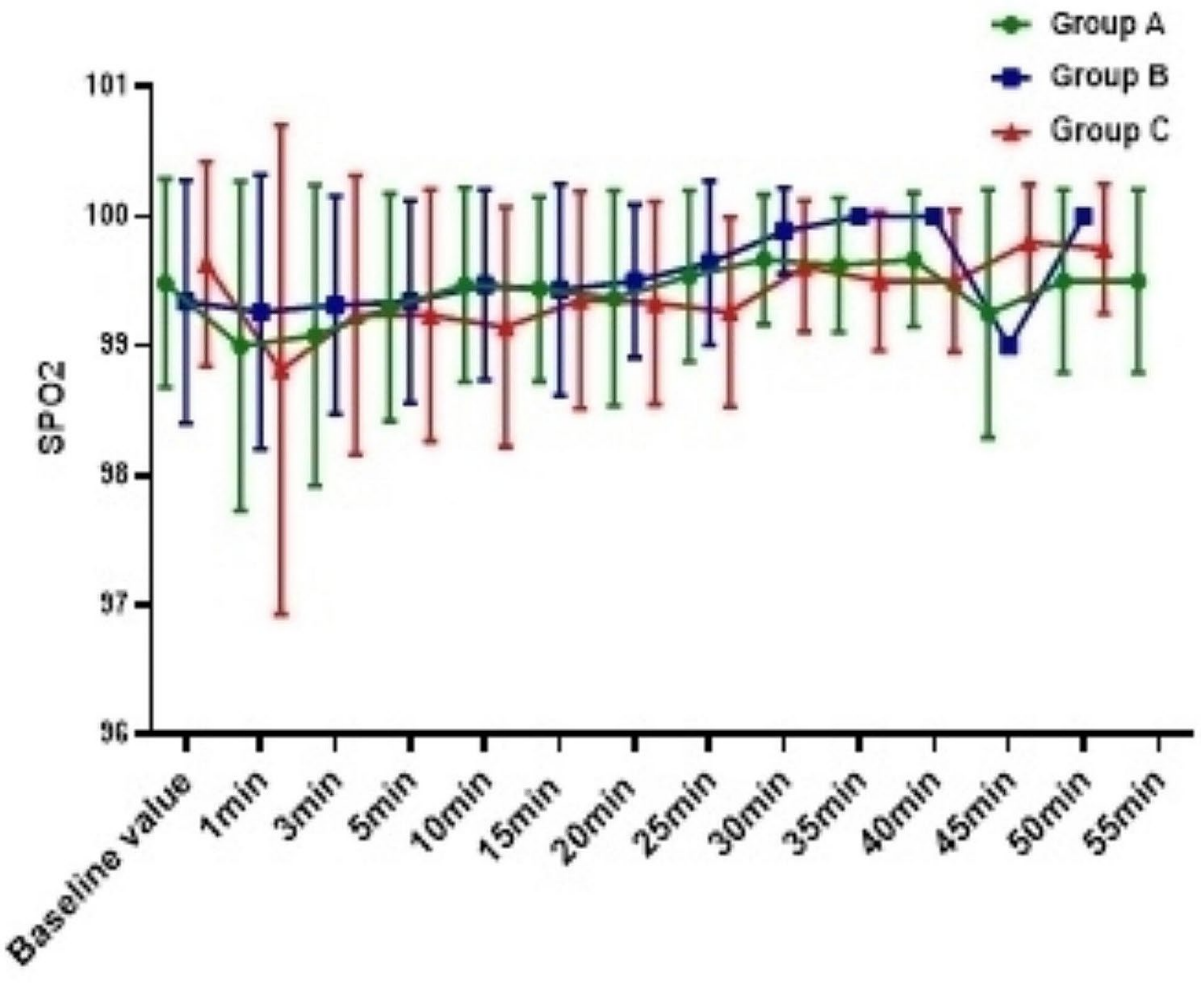
The change trend of SpO_2_ at each time point during the perioperative period was compared among the three groups

**Fig. 6:**
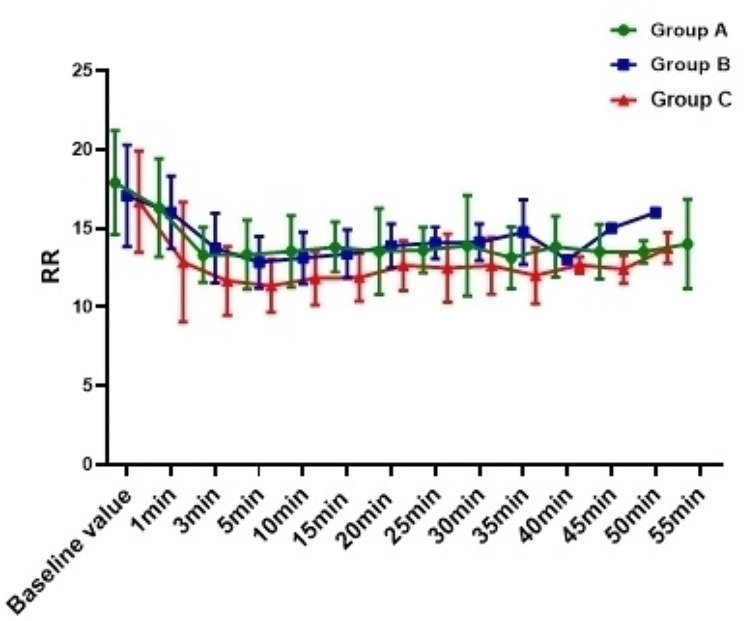
The change trend of RR at each time point during the perioperative period was compared among the three groups

The incidence of hypotension in Group C was 54.0%, which was significantly higher than Group A (10.8%) and Group B (18.9%) ( *P*<0.001), and there was no difference between Group A and Group B (*P* = 0.744); In addition, compared with Group C, the overall fluctuation range of blood pressure in Group A and Group B was smaller (Figs. 2, 3 and 4). The incidence of bradycardia in Group A(5.4%) and B (5.3%) was significantly lower than that in Group C (18.9%, *P* = 0.107); The heart rate diachronic analysis showed that the heart rate fluctuation of Group A and Group B was smaller than that of Group C (Fig. 2); There was no hypoxemia in Group A, the incidence of hypoxemia in Group B was significantly lower than that in Group C (5.3% vs. 10.5%, *P* = 0.163); The diachronic analysis of SpO_2_ and RR (Figs. 5 and 6) showed that the respiratory inhibition of Group A and Group B was smaller than that of Group C.

### Adverse events

**Fig. 7:**
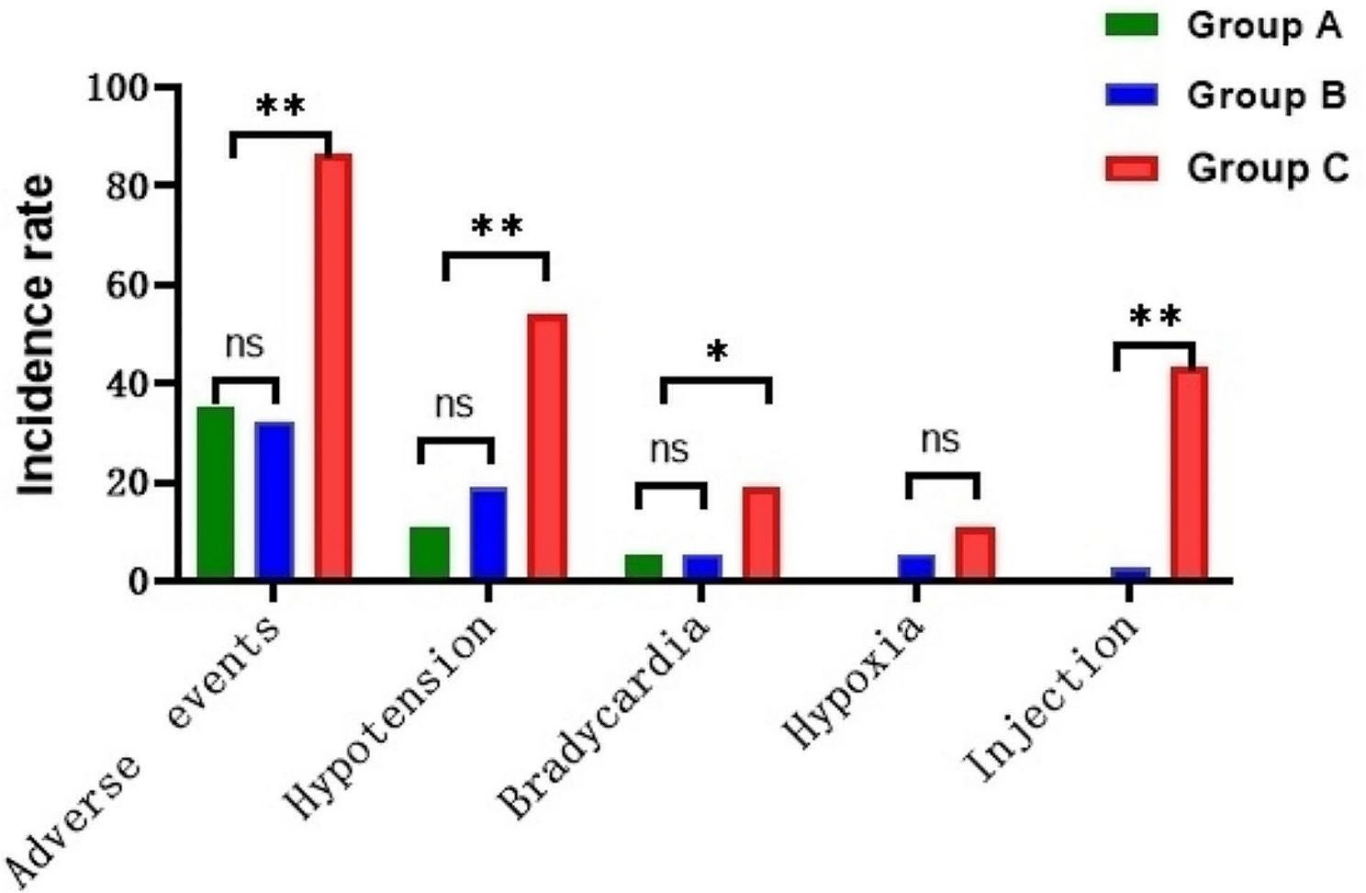
The rates of major adverse reactions during perioperative period were compared among the three groups

After administration, adverse events occured in Group C was 71.0% higher than Group A (27.0%) and Group B (36.8%) (*P*<0.001) with no life threatening events or deaths; The degree of pain at the injection site in three groups was ≤ 2, and the incidence of pain at the injection site in Group A (not occurred) and Group B (2.6%) was lower than Group C(42.1%) (*P*<0.001) (Table 7; Fig. 7).

**Table 7:** Incidence of treatment-emergent adverse events

	Group A (*n* = 37)	Group B (*n* = 38)	Group C (*n* = 38)	*P* value
Any treatment-emergent adverse events	10/37(27.0%)	14/38(36.8%)	27/38(71.0%)	*P*<0.001
Hypertension	2/37(5.4%)	2/38(5.3%)	1/38(2.6%)	0.870
Hypotension	4/37(10.8%)	7/38(18.4%)	20/38(52.6%)	*P*<0.001
Tachycardia	0/37(0%)	1/38(2.6%)	0/38(0%)	1.000
Bradycardia	2/37(5.4%)	2/38(5.3%)	7/38(18.4%)	0.107
Hypoxia	0/37(0%)	2/38(5.3%)	4/38(10.5%)	0.163
Dizziness	3/37(8.1%)	1/38(2.6%)	1/38(2.6%)	0.447
Hiccup	1/37(2.7%)	1/38(2.6%)	0/38(0%)	0.772
Injection pain	0/37(0%)	1/38(2.6%)	16/38(42.1%)	*P*<0.001
Obvious body movement	4/37(10.8%)	2/38(5.3%)	0/38(0%)	0.083
Obvious peristalsis	3/37(8.1%)	0/38(0%)	0/38(0%)	0.033

### Successful sedation

The sedation success rate was similar at 90.2%, 92.7% and 92.7% in Group A, B and C, respectively (*P* = 1.000). Propofol and sufentanil were used as rescue drugs for failure of sedation (Table 8).

There are 4 cases failure of sedation in group A, and these patients were sedated quickly after the first administration, with MOAA/S ≤ 3 (2 males and 2 females); Three of patients had obvious body movement during the operation of doctors, which affected the operation. The body movement reaction of the patients was still obvious after two consecutive drug supplementation, and then they were sedated with other sedatives. Another case showed obvious intestinal peristalsis during the operation of the digestive endoscopist, and the endoscopist complained that the patient had intestinal spasm and required propofol to complete sedation.

There are 3 cases failure of sedation in group B, and all patients were sedated quickly after the first administration, with MOAA/S ≤ 3 (2 male and 1 female); Two of patients had obvious body movement during the operation of digestive endoscopy doctors, which affected the operation, and their body movement was still obvious after two consecutive drug additions, and propofol was then used to maintain sedation. In another case, the doctor considered that the patient had obvious intestinal distortion and high difficulty during the operation of digestive endoscopy, so other drugs were required to maintain sedation.

There were 3 cases of sedation failure in group C, all patients were sedated quickly after the first administration, and the MOAA/S ≤ 3 (3 women). 2 patients showed obvious body movement during the operation doctors, and the obvious body movement was still seen after two consecutive doses of 30–50 mg propofol, and then sufentanil and propofol were added to maintain sedation level. The other patient showed MOAA/S > 3 points during the operation of doctor, and the MOAA/S > 3 points remained after the addition of 50 mg of propofol for two consecutive times, and the subsequent addition of propofol to maintain sedation level.

**Table 8:** Sedation success rate in 3 groups (%)

Index	Group A (*N* = 37)	Group B (*N* = 38)	Group C (*N* = 38)	*P*
Sedation success rate	37/41(90.2%)	38/41(92.7%)	38/41(92.7%)	1.000

### Intraoperative sedation

**Fig. 8:**
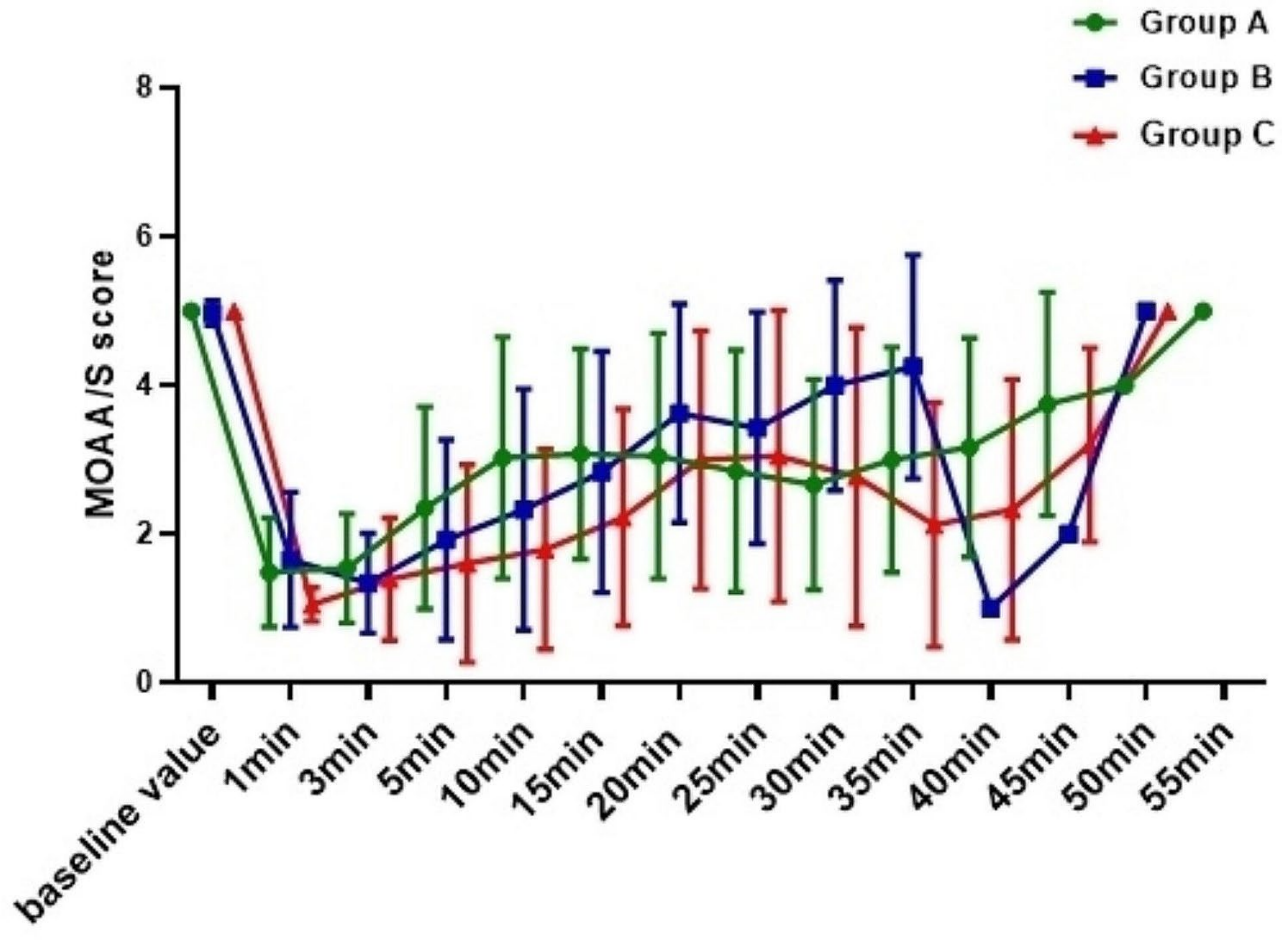
The change trend of MOAA/S at each time point during the perioperative period was compared among the three groups

**Fig. 9:**
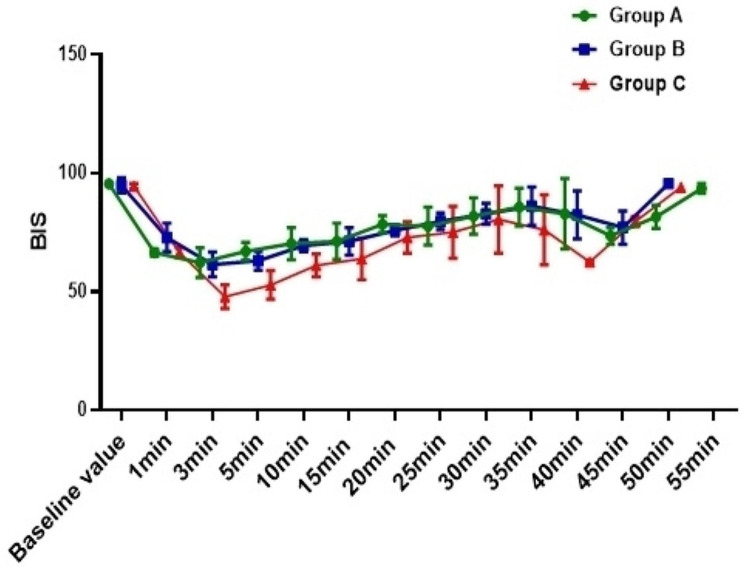
The change trend of BIS at each time point during the perioperative period was compared among the three groups

At T1, the MOAA/S scores of Group C were significantly lower than those of group B (*P*<0.001); At T1, T3 and T4, the MOAA /S scores of Group C were significantly lower than those of Group A (*P* = 0.001, *P* = 0.001, *P*<0.001); There was no statistically significant difference in MOAA/S scores between Group A and Group B at T0,T1,T2,T4,T5 (*P* = 1.000, *P* = 1.000, *P* = 0.193, *P* = 0.073,*P* = 1.000) (Table 9; Fig. 8). Timepoints T2, T3 and T4 are showing lower BIS values in Group C than those in Group A and Group B (T2:*P*<0.001, *P*<0.001; T3:*P* = 0.003, *P* = 0.033; T4:*P*<0.001, *P* = 0.002) (Fig. 9). (Due to the limitation of reality, only some subjects in each group were selected for BIS monitoring).

**Table 9:** Comparison of MOAA/S score at each observation time point

	Group A(*N* = 37)	Group B(*N* = 38)	Group C(*N* = 38)	*P* value	Comparison
T0	5.0 ± 0.00	5.0 ± 0.00	5.0 ± 0.00	*P* = 1.000	
T1	1.5 ± 0.73	1.7 ± 0.91	1.1 ± 0.23	*P*<0.001	Group C vs. Group A: *P* = 0.001 Group C vs. Group B: *P*<0.001 Group A vs. Group B: *P* = 1.000
T2	1.5 ± 0.73	1.3 ± 0.67	1.4 ± 0.82	*P* = 0.193	
T3	2.4 ± 1.32	1.9 ± 1.34	1.6 ± 1.33	*P* = 0.002	Group C vs. Group A: *P* = 0.001 Group C vs. Group B: *P* = 0.170 Group A vs. Group B: *P* = 0.028
T4	3.1 ± 1.66	2.5 ± 1.69	2.0 ± 1.50	*P* = 0.007	Group C vs. Group A: *P*<0.001 Group C vs. Group B: *P* = 0.170 Group A vs. Group B: *P* = 0.073
T5	5.0 ± 0.00	5.0 ± 0.00	5.0 ± 0.00	*P* = 1.000	

### Awakening and recovery

The mean time to reach post anaesthesia care unit discharge criteria was shorter for Group A (16.2 min) and Group B (16.5 min) as compared to Group C (19.5 min) (*P* = 0.011); There was no significant difference between Group A and Group B (16.2 min vs 16.5 min, *P* = 0.742)(Table 10).

**Table 10:** Mean times for recovery (minutes)

		Group A (*N* = 37)	Group B (*N* = 38)	Group C (*N* = 38)	*P* value
From last study medication to fully alert	Mean	8.8	9.2	8.2	0.443
	SD	3.92	4.04	3.50	
From last study medication to Modified Aldrete score > 9	Mean	16.2	16.5	19.6	0.011
	SD	4.45	3.65	5.67	

### Drug usage in test groups

**Fig. 10:**
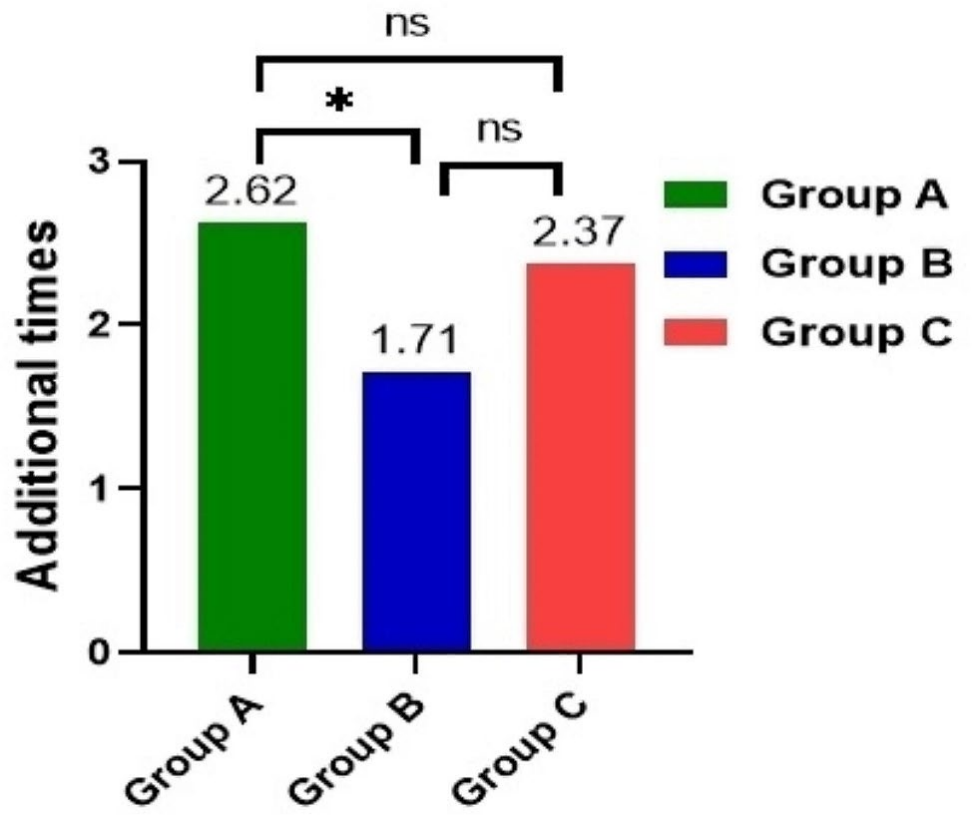
The perioperative sedative drugs were compared among the three groups

**Fig. 11:**
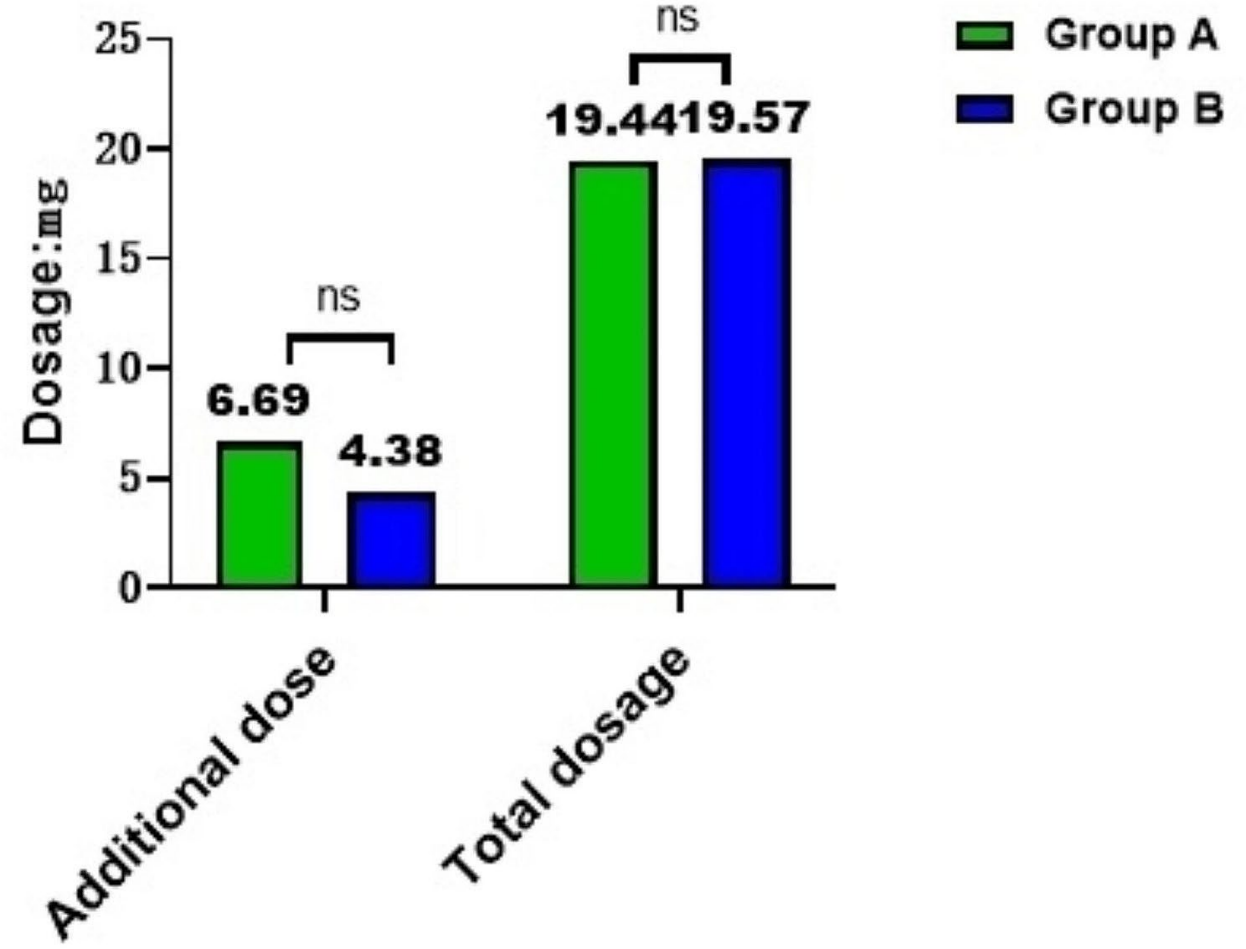
Comparison of the first dose, additional dose and total dose of remimazolam between two groups

The average first dose of patients in Group A was lower than Group B, while the average number of additions was higher than Group B (*P* = 0.022), and there was no significant difference between Group A and Group C or between Group B and Group C (*P* = 0.584, *P* = 0.867) (Fig. 10) (Fig. 11). However, there was no significant difference between the average supplemental dose and the average total amount of medication in group A and group B (*P* = 0.469) (Fig. 10) (Table 11).

**Table 11:** Supplemental interval and frequency of remimazolam supplementation

		Group A (*N* = 37)	Group B (*N* = 38)	*P* value
First addition interval (min)	Mean	5.5	7.3	0.469
	SD	2.7	3.4	
Add times (times)	Mean	2.6	1.7	0.022
	SD	2.0	1.4	

## Discussion

This study showed that the sedation success rate in the remimazolam group was similar to that in the propofol group (*P* = 1.000). Compared with propofol, remimazolam showed shorter time to fully alert and reach post anesthesia care unit (PACU) discharge criteria. The MOAA/S scores of the three groups were ≤ 3 min after administration 1 min, indicating that the induction dose of remimazolam and propofol enable subjects to achieve the target sedation depth in a short time.

There was no statistical difference between the three groups in the average time from the last administration to full alert, indicating that the inhibition of remimazolam and propofol on the central nervous system was reversible, and the recovery time was not significantly different. The time from the last administration to reach post-anesthesia care unit discharge criteria of Group A and Group B was significantly shorter than Group C (*P* = 0.011), indicating that the metabolism time of remimazolam was shorter than propofol. This advantage is attributed to the molecular design of remimazolam. Its ultra-short-acting properties lead to its rapid breakdown into inactive metabolites by ubiquitous tissue esterase. On the other hand, this advantage was attributed to the lower depth of sedation induced by remimazolam. The sedation curve showed that the propofol group had deeper sedation and longer recovery time than the remimazolam group. This is consistent with its properties as a benzodiazepine [21]. Also, colonoscopy is a short procedure that does not require such a depth of sedation. Thus, remimazolam is sufficient to provide an acceptable sedation protocol for colonoscopy.

In the present study, remimazolam was used as a sedative agent in combination with sufentanil to achieve the level of sedation required for the colonoscopy without prolonging the time to full alert and the time to discharge criteria. Our results were also consistent with those of previous studies in showing that remimazolam was well tolerated and non-inferior to propofol [22–24].

The adverse events rate of Group C was significantly higher than that of Group A and Group B (*P*<0.001) indicated that remimazolam has less circulatory inhibition and higher safety. Colonoscopy requires intestinal preparation and fasting the day before, which makes the patient’s effective circulating blood volume relatively insufficient, and propofol lower blood pressure more significantly through the dual effects include peripheral vascular expansion and myocardial inhibition [25, 26].Therefore, we need drugs with less influence on circulation to make the sedation process more secure.

Compared with propofol, remimazolam showed a significantly lower incidence of hypotension and hypoxemia. Respiratory and circulatory depression is the most common presentation after brain stem inhibition by narcotic drugs [27]. R. N. Upton*et al. think that high doses of CNS7056 (remimazolam) and propofol cause short-term respiratory and circulatory depression of similar magnitude and duration [28]. Therefore, the small inhibitory effects of remimazolam on respiration and circulation in this study may be due to the relatively small dosage of remimazolam, or it may be due to its unique pharmacological structure, which has a relatively mild inhibitory effect on the brain stem.

The incidence of pain at the injection site in Group A and Group B was lower than Group C (0 vs. 2.6% vs42.1%), and the degree of injection pain in Group B was grade 1, which showed that remimazolam effectively reduced the incidence of injection pain. This may be attributed to the fact that remimazolam is configured with normal saline and its components provide no or less stimulation of the vessel wall. Thus, remimazolam is sufficient to provide an acceptable sedation protocol for colonoscopy.

In treatment groups, it was divided into Group A and Group B according to the first dosage. It showed that 0.20 mg /kg and 0.25 mg /kg remimazolam could achieve satisfactory sedation effect. However, remimazolam as an ultra-short-acting sedative, has too short a duration of action, which results in its rapid metabolism during injection and therefore requires a large supplementary dose during endoscopy [29, 30]. Compared with the Group A, Group B had significantly less additional times and additional volume, and did not increase the incidence of adverse reactions. The 0.3 mg/kg dosing group that was available in the study for observation and comparison study was not selected in this study because the depth of sedation was similar to that of the first two groups when induction was performed at that dose in the preliminary study. The recovery time was slightly prolonged. There is little need for additional medication.Considering that we need patients to recover quickly and accurately control the dose of drugs for painless colonoscopy, this group was not set as the experimental group. Thus, the induction dose of remimazolam from 0.2 mg/kg to 0.25 mg/kg with 2.5 mg /kg additional dose is safe and effective.

In addition, 2 cases of intractable hiccup occurred within 2–3 min after the administration of remimazolam in this study. The possible reasons as follows: (a) The reaction caused by pharmacological effects of remimazolam or sufentanil or additives; (b) The reaction caused by the patient’s own tension; (c) Adverse reactions caused by endoscopic operation or air inflation to stimulate the intestine. Hiccup was relieved gradually when patients recovered to fully alert, without obvious discomfort; The published research has not found the explanation of this adverse reaction, so we should be cautious.

In addition, our study was limited by its sample size, single blind design and the age of the subjects. The cases are all from the data of a single center. Larger population and multi-center studies are needed to provide reference for painless colonoscopy.

## Conclusions

In this trial, remimazolam (Remimazolam besylate) provided safe and effective sedation for colonoscopy and reduced the occurrence of adverse reactions such as hypotension, respiratory depression, and injection pain. The initial dose of remimazolam of 0.25 mg/kg and the supplementary dose of 2.5 mg/ kg can achieve satisfactory sedation induction and maintenance effects.

## Data Availability

All data generated or analysed during this study are included in this published article. Some of the raw data are not publicly available and are available from the authors on request.

## Abbreviations

ASA: American Society of Anesthesiologists
BIS: Bispectral index
BMI: Body Mass Index
DBP: Diastolic blood pressure
GABA: γ-aminobutyric acid
HR: Heart rate
MAP: Mean arterial pressure
MOAA/S: Modified observer’s assessment ofalertness /scores
NIBP: Non-invasive blood pressure
NS: Normal saline
RR: Respiratory rate
SBP: Systolic blood pressure
SPSS: Statistic Package for Social Science
SpO_2_: Pulse oxygen saturation
VRS: VerbalRating Scale
